# Waist-to-calf circumstance ratio and cognitive function among Chinese older adults: Mediating roles of physical performance and social activity

**DOI:** 10.3389/fnagi.2023.1166341

**Published:** 2023-04-17

**Authors:** Xia Cao, Binfang Yang, Jiansong Zhou

**Affiliations:** ^1^Health Management Center, The Third Xiangya Hospital, Central South University, Changsha, China; ^2^Hunan Chronic Disease Health Management Medical Research Center, The Third Xiangya Hospital, Central South University, Changsha, China; ^3^Department of Psychiatry, The Second Xiangya Hospital, Central South University, Changsha, China; ^4^National Clinical Research Center for Mental and Mental Diseases, The Second Xiangya Hospital, Central South University, Changsha, China

**Keywords:** waist-to-calf circumstance ratio, cognitive function, physical performance, social activity, sarcopenic obesity, mediation analysis

## Abstract

**Background:**

In light of the potentially detrimental effects of central fat and decreased muscle mass on cognitive function, it would be beneficial to learn more about the mediating mechanisms underpinning the association between the two. The purpose of this study is to determine the association between waist-to-calf circumstance ratio (WCR) and cognitive function, as well as to investigate whether physical performance and social activity mediate the relationship between WCR and cognitive function among older Chinese adults.

**Methods:**

An analysis of 9,652 older Chinese adults was conducted during the 2018 wave of the Chinese Longitudinal Health Longevity Survey (CLHLS). The Mini-Mental State Examination (MMSE) and a self-reported scale were used to measure cognitive function, physical performance, and social activity, respectively. Multiple linear regression and mediation analyses were conducted.

**Results:**

The findings suggest that a high WCR had a significant negative association with cognitive function (*B* = −0.535, 95% CI: −0.754, −0.317). Mediation analysis revealed that a high WCR influenced old adults' cognitive function in three ways: first, through the partial mediating effect of physical performance (*B* = −0.270; 95% CI: −0.340, −0.203); second, through the partial mediating effect of social activity (*B* = −0.035; 95% CI: −0.055, −0.017); and third, through the serial mediating effects of physical performance and social activity (*B* = −0.021, 95% CI: −0.029, −0.015).

**Conclusion:**

The study results suggest the adverse impact of a high WCR on older adults' cognitive function, and the possible mechanisms of physical performance and social activity by which the association takes place. Multidimensional health and social interventions aimed at improving physical, social, and cognitive functioning among older adults with sarcopenic obesity are recommended.

## 1. Introduction

Attention to variance in health, physical and cognitive function, and social embeddedness in a varied and aging community is critical to successful aging. According to the World Health Organization, ~10 million people with cognitive impairments around the world develop dementia each year and more than 50 million people are diagnosed with it (Abdivalievna, 2022). A recent meta-analysis revealed that the global prevalence of mild cognitive impairment was 15.56% (95% CI: 13.24–18.03%) in community-dwelling adults aged 50 years and older (Atkins and Wannamathee, 2020). As the largest developing country, China is becoming an aging society. Almost one-fifth of China's population (18.70% in 2020) is over 60 years, and 13.50% is over 65 years. Mild cognitive impairment to severe dementia is common among older adults over 65 years old, accompanied by a high burden of limited life expectancy and healthcare utilization (Auyeung et al., 2013). According to a nationally representative survey in China, the prevalence of dementia was 6.0% and that of cognitive impairment was 15.5% among adults aged 60 years and older, representing 15.07 million people with dementia and 38.77 million people with cognitive impairment (Bai et al., 2022). Not only in China but also globally, large dementia and cognitive impairment population has become a significant health burden, necessitating stronger antidementia measures to combat this disease (Barnett et al., 2012). It is, therefore, imperative to identify and manage modifiable risk factors associated with cognitive impairment.

Previously known risk factors for cognitive impairments include age, sex, hypertension, diabetes mellitus, hyperlipidemia, stroke, congestive heart disease, chronic renal failure, homocysteinemia, and poor lifestyle (Batsis and Villareal, 2018). Meanwhile, obesity and sarcopenia, two important public health issues among older adults worldwide, are associated with a variety of risk factors that negatively affect long-term cognitive function (Berkman et al., 2000; Bennett et al., 2006; Batsis et al., 2021; Bilski et al., 2022). Whereas, sarcopenic obesity (SO) acts as a new category of obesity as well as a high-risk geriatric syndrome, and less is known about the effect of SO on cognitive performance in elderly individuals (Brewster et al., 2021). Although there is no consensus regarding the definition of SO (Brown et al., 2016), the coexistence of low muscle mass and strength as well as excess adiposity is this core connotation (Cauley, 2015; Buie et al., 2019). The prevalence of SO has risen in recent years due to the aging population crisis. Due to the lack of a uniform definition for SO and different study populations, its reported prevalence estimates range from 2.75% to 20% or more (Chang et al., 2015). According to some previous reports, the prevalence of SO in Chinese community-dwelling older adults was estimated at 6.0–25.0% (Chang et al., 2016; Chen et al., 2021; Cheng et al., 2022). SO is associated with poor health outcomes including frailty, disability (Choe et al., 2018), fractures, falls (Chou et al., 2022), cancer (Daviglus et al., 2010), cardiometabolic diseases (Donini et al., 2020), chronic kidney disease (Dye et al., 2017), and increased mortality (Espeland et al., 2022). Those who suffer from this geriatric syndrome are at risk of synergistic complications that can eventually result in long-term functional decline. Of importance, studies have shown associations between SO and impaired mental health (Etgen et al., 2011; Chen et al., 2021; Fan et al., 2021), including depression, dementia, and cognitive decline, as well as decreased psychological wellbeing. Considering these associations, researchers and policy experts are increasingly interested in identifying interventions that could improve health outcomes for older adults with SO.

Despite a few reports on the association between SO and cognition among old adults (Gao et al., 2015, 2020), sufficient attention has not been paid to cognitive changes in older adults with SO. In light of the potentially detrimental effects of SO on older adults' cognitive function, it is important to develop an understanding of the mediating mechanisms underlying this association. It would be helpful to identify these mechanisms to provide appropriate prevention and interventions to older adults who are confounded by SO. There is evidence that SO may have a synergistic effect on energy balance and muscular function/physical capacity (Gao et al., 2022). Based on some observations (Hayes, 2012; Hayes and Preacher, 2014; Henn et al., 2022), low levels of physical activity or physical fitness were observed in individuals with SO. Meanwhile, older persons with long-term functional impairments who engage with numerous barriers may have equal access to adequate social services (Hirani et al., 2017). Cognitive decline is less pronounced in socially active older adults in late life (Hirani et al., 2017). However, the underlying role of social activity in mediating the interaction of SO and cognitive function among elderly individuals with SO has not received full attention. Since SO has become a significant health concern among older adults and is greatly undertreated (Buie et al., 2019), exploring its influence on cognitive function and its potential social-psychological mediating factors is an appealing and potentially influential strategy to promote healthy aging. Mild cognitive impairment to severe dementia is common among elderly individuals over 65 years old, accompanied by a high burden of limited life expectancy and healthcare utilization (Auyeung et al., 2013). In light of the potentially detrimental effects of SO on mental health and cognition in older adults, a better understanding of the underlying mechanisms behind SO and cognitive decline is crucial. It would be helpful to identify these mechanisms to provide appropriate prevention and interventions to older adults facing age-related changes in body composition and physical dysfunction. Most importantly, studies have been rarely conducted in which both psychological and social pathways have been studied simultaneously to understand how SO affects cognitive function.

Therefore, to fill the abovementioned gaps, we chose the representative elderly subject from the Chinese Longitudinal Healthy Longevity Survey (CLHLS) database, since those included elderly population aged 65 years and older are reported to be susceptible to SO, as well as exhibiting an elevated level of cognitive impairment (Ida et al., 2018; Hong, 2020), with a diverse range of observed variables (Ji et al., 2022). In this study, we propose a higher adiposity-to-muscle ratio, that is high waist-to-calf circumstance ratio (WCR, see later), as an anthropometric measure of SO. As an index assessing the disproportion between abdominal fat and leg muscle mass, WCR has served as an alternative measure of SO in several studies (Jia et al., 2020, 2022; Jo et al., 2022). The first aim of this study was to examine the relationship between WCR and cognitive function among older Chinese adults based on a database. A further objective of the study was to explore the role of physical performance and social activities in moderating the relationship between WCR and cognitive function. Specifically, we proposed the following hypotheses. First, there is a relationship between WCR and cognitive function among elderly individuals in China. Second, physical performance and social activities would mediate the association between WCR and cognitive function. Finally, physical performance and social activities would have a serial mediation effect between WCR and cognitive function (Supplementary Figure S1).

## 2. Methods

### 2.1. Study design and participants

We conducted a secondary analysis of the dataset derived from CLHLS-2018, a nationally representative prospective cohort study that recruited adults aged 65 years and older in major provinces of China. Details of the study participants and methods have been reported elsewhere (Kelly et al., 2017; Katayama et al., 2022). The CLHLS 2018 interviewed 15,874 older adults with a standard questionnaire using face-to-face interviews. Before the survey, each participant or proxy respondent signed an informed consent form. The research was approved by the Research Ethics Committee of Peking University (approval number: IRB00001052-13074). Those younger than 65 years of age (n = 95) were excluded from the current analysis. We restricted our final analysis to 9,652 older adults with completed information on the questions we are concerned about. Details of the screening procedure are described in Supplementary Figure S2. Based on existing research (Kohara et al., 2017; Kokkeler et al., 2019; Kim and Yoon, 2022), along with the design of the CLHLS questionnaire, a set of variables was selected for analysis (Supplementary Table S1).

### 2.2. Dependent variables

Cognitive function. Based on previous studies, this study used the Chinese version of a modified MMSE to measure cognitive function (Levine and Crimmins, 2012; Katayama et al., 2022), scoring ranging from 0 to 30, with a lower score indicating poorer cognitive performance. It includes 24 items regarding orientation, attention, registration, calculation, recall, and language. Cronbach's α coefficient of the MMSE for this study was 0.91. More details about this scale can be found in Supplementary Table S2.

### 2.3. Independent variables

High waist-to-calf circumstance ratio. Participants in CLHLS-2018 were instructed to relax their bodies before a measuring tape was used to measure their waist circumference (WC) and calf circumference (CC). WC was measured at the midpoint between the lower ribs and iliac crest at the end of expiration. CC was measured at the site of the largest circumference of the right calf of each participant while seated. WCR was the ratio of the two (Li et al., 2022). In this study, independent variables were created by assigning participants to tertiles of WCR. The highest tertile of WCR was classified as high WCR, while the lowest and middle tertiles of WCR were classified as low to intermediate WCR.

### 2.4. Mediators

Physical performance. According to a recent study (Kim and Yoon, 2022), muscle strength, walking ability, the strength of the lower extremities, and core strength were included in the present study. The relevant entries were collected in our study based on four questions (scoring range from 0 to 8), as described in Supplementary Table S3. A higher total score indicated poor physical performance. Cronbach's α coefficient of the score for this study was 0.87 (Kim and Yoon, 2022).

Social activity. In the context of the framework of Liu et al. (2022), playing cards/mahjong, participating in organized social activity, and visiting experiences were included in the present study, under the subdomain of social activity (Ma et al., 2022). According to previous studies, the relevant entries were collected in our study based on three questions (scoring range from 0 to 3), as described in Supplementary Table S4.

### 2.5. Covariates

As covariates, sociodemographic characteristics, lifestyles, and health status were classified as potentially related factors in previous studies (Levine and Crimmins, 2012; Kokkeler et al., 2019).

Sociodemographic characteristics. The sociodemographic characteristics included age (in years), sex (male or female), education (illiterate or literate), residence (rural or urban), marital status (married and living with a spouse or other statuses), living arrangement (living alone or living with someone else), occupation before age 60 (non-professional work or professional work), and financial support (insufficient or sufficient).

#### 2.5.1. Lifestyle

As described in a recent study (Mendham et al., 2021), by adding the dietary pattern and daily habit scores, a combined lifestyle score was calculated between 10 and 50, as described in Supplementary Table S5. Since the missing proportion of the collected data in this domain was <5%, the average scores of each variable were utilized to replace the missing values. For analysis, the eight frequency options of food intake were measured, including staple foods, meat, fish, fresh fruits and vegetables, milk, sugar, and nuts (Mo et al., 2022). The dietary pattern score is determined as the total of all eight food category scores, ranging from 7 to 38, with higher scores reflecting better eating patterns, as indicated in prior research (Moreno-Franco et al., 2018; Mendham et al., 2021; Mo et al., 2022). Tobacco and alcohol consumption as well as the amount of outdoor exercise were also recalled by participants. The scores ranged from 1 to 4 for tobacco use and 1 to 3 for alcohol consumption. A higher score indicated fewer daily smoking or drinking sessions (Mendham et al., 2021). The participants were asked to rate how often they performed outdoor activities, with a score ranging from 1 to 5 in ascending order according to the frequency of their participation. Across all daily life habits, the score ranged from 3 to 12 (Mendham et al., 2021).

#### 2.5.2. Health status

This study evaluates health status primarily through body mass index (BMI, continuous variable), physical comorbidity of chronic diseases, and depressive symptoms. Physical comorbidity was measured by measuring 13 chronic conditions (e.g., hypertension, diabetes, stroke, cancers, Parkinson's disease) in the CLHLS (Panickar and Jewell, 2015; Ozkok et al., 2022). Physical comorbidities were defined as self-reported diseases or conditions that exceeded two of the 13 listed earlier (Panickar and Jewell, 2015; Ozkok et al., 2022). The 10-item Center for Epidemiologic Studies Depression (CES-D-10) was used to assess depressive symptoms, which was a self-reported scale for assessing the symptoms of depression in the past week (Peng et al., 2020). The CES-D-10 contains 10 items on somatic symptoms, depression impacts, and positive affect. In each item, a score is assigned between 0 and 3 (“rarely” to “almost always”), as described in Supplementary Table S6. The total score ranges from 0 to 30 with higher total scores indicating more severe depressive symptoms. A score of 10 or higher indicates possible depression. The CES-D-10 has been validated among older adults in China (Peng et al., 2020; Picca et al., 2022). Cronbach's α coefficient of the CES-D-10 for this study was 0.87.

### 2.6. Statistical analysis

For summary statistics, numerical variables are represented as the means and standard deviations, whereas categorical data are provided as frequencies. To assess differences between proportions and means, chi-square tests and *t*-tests were used. First, multivariate linear regression analysis was performed to explore the relationships between high WCR and cognitive function. Then, as determined by B-coefficients with 95% confidence intervals (CIs) from the initial analyses, significance for the next interaction analyses was set at *P* < 0.10. Finally, the PROCESS macro for SPSS was adopted to examine the mediation model (Pindus et al., 2021). In the Hayes PROCESS, the coefficients of the conditional indirect effects and conditional mediator tests are estimated along with the bias-corrected bootstrap confidence intervals. The regression-based, path-analytic framework we used in our analysis to determine if there was a serial mediation effect of physical performance and social activity between high WCR and cognitive function in older adults, relevant methods can be found in previous studies (Polyzos and Margioris, 2018). The mediation's significance was determined by computing bias-corrected 95% confidence intervals (CIs) with bootstrapping (5,000 resamples) (Preacher et al., 2007). In those models, covariates included age, sex, education level, residence, marital status, living arrangement, occupation before age 60, financial support, lifestyle, BMI, physical comorbidities, and CES-D-10 score. All analyses were conducted in IBM SPSS 24.0.

## 3. Results

### 3.1. Sample characteristics

As shown in Table 1, we compared the sample characteristics stratified by WCR. Among the 9,652 participants (4,588 men, 5,064 women), 2,837 (29.39%) had a high WCR (upper WCR tertile). Participants with a high WCR had a lower average MMSE score than those with low to intermediate WCR (24.09 vs. 26.28, *P* < 0.001). Those with high WCR were generally older, female, with lower levels of education, living in a rural area, widows/separated/single, living alone, with non-professional work before retirement, with lower lifestyle score, with lower BMI, and having a higher average CES-D-10 score (*P* < 0.05 or *P* < 0.001). Compared to those with a low to intermediate WCR, those with high WCR had a higher average physical performance score (2.85 vs. 1.74, *P* < 0.001) as well as a lower average social activity score (0.80 vs. 1.06, *P* < 0.001). Neither group showed significant differences regarding financial support or physical comorbidities (*P* > 0.05).

**Table 1:** Characteristics of the sample stratified by waist-to-calf circumstance ratio (WCR).

**Characteristics**	**Total Mean ±SD or *N* (%)**	**Low to intermediate WCR Mean ±SD or *N* (%)**	**High WCR Mean ±SD or *N* (%)**	***χ^2^*** or ***t*** **statistics**	***P*** **-value**
*N*	9,652	6,815	2,837	–	–
Age (years)	83.09 ± 10.95	81.59 ± 10.60	86.69 ± 10.94	−21.33	<0.001
**Sex**					
Male	4,588 (47.5)	3,629 (53.3)	959 (33.8)	303.76	<0.001
Female	5,064 (52.5)	3,186 (46.7)	1,878 (66.2)		
**Education level**					
Illiterate	4,928 (51.1)	3,128 (45.9)	1,800 (63.4)	246.86	<0.001
Literate	4,724 (48.9)	3,687 (54.1)	1,037 (36.6)		
**Residence**					
Rural	4,720 (70.8)	4,720 (69.3)	2,109 (74.3)	24.98	<0.001
Urban	2,095 (29.2)	2,095 (30.7)	728 (25.7)		
**Marital status**					
Married	4,566 (47.3)	3,564 (52.3)	1,002 (35.3)	231.62	<0.001
Other statuses	5,086 (52.7)	3,251 (47.7)	1,835 (64.7)		
**Living arrangement**					
Living alone	1,535 (15.90)	1,044 (15.3)	491 (17.3)	5.92	0.015
Living with someone else	8,117 (84.10)	5,771 (84.7)	2,346 (82.7)		
**Occupation before age 60**					
Non-professional work	8,564 (88.7)	5,951 (87.3)	2,613 (92.1)	45.80	<0.001
Professional work	1,088 (11.3)	864 (12.7)	224 (7.9)		
**Financial support**					
Insufficient	1,301 (13.5)	933 (13.7)	368 (13.0)	0.89	0.346
Sufficient	8,351 (86.5)	5,882 (869.3)	2,469 (87.0)		
Total lifestyle score	29.91 ± 4.76	30.04 ± 4.81	29.60 ± 4.62	4.19	<0.001
Body mass index (kg/m^2^)	23.53 ± 7.02	23.97 ± 6.89	22.47 ± 7.20	9.61	<0.001
**Physical comorbidities**					
Yes	3,308 (34.3)	2,306 (33.8)	1,002 (35.3)	1.95	0.162
No	6,344 (65.7)	4,509 (66.2)	1,835 (64.7)		
CES-D-10 score	7.33 ± 4.43	7.20 ± 4.43	7.65 ± 4.40	−4.57	<0.001
Physical performance score	2.06 ± 2.38	1.74 ± 2.23	2.85 ± 2.55	−21.47	<0.001
Social activity	0.98 ± 0.83	1.06 ± 0.85	0.80 ± 0.77	14.61	<0.001
MMSE score	25.64 ± 5.76	26.28 ± 5.17	24.09 ± 6.74	17.30	<0.001

Comparison was performed using the t-test or Chi-square test. SD, standard deviation. CES-D-10, The 10-item Center for Epidemiologic Studies Depression. MMSE, mini-mental state examination.

### 3.2. The association between WCR and cognitive function

In Table 2, we examined the unadjusted association between WCR and cognitive function (Model 1). WCR and cognitive function exhibited a significant negative correlation (*B* = −2.194, 95% CI: −2.443, −1.946). After adjusting for sociodemographic characteristics, lifestyles, and health status (Model 2), this correlation remained present (*B* = −0.533, 95% CI: −0.752, −0.315). Age (*B* = −0.216, 95% CI: −0.227, −0.205), sex (*B* = −0.805, 95% CI: −1.028, −0.583), and CES-D-10 score (*B* = −0.165, 95% CI: −0.187, −0.143) were negatively correlated with cognitive function. However, the education level (*B* = 1.051, 95% CI: 0.829, 1.274), marital status (*B* = 0.256, 95% CI: 0.022, 0.490), professional work before retirement (*B* = 0.480, 95% CI: 0.149, 0.810), BMI (*B* = 0.027, 95% CI: 0.011, 0.043), and higher total lifestyle score (*B* = 0.081, 95% CI: 0.059, 0.103) showed significantly positive correlations with cognitive function. The direction or significance of the associations did not transform substantially from Model 1 to Model 2, which adjusted for covariates.

**Table 2:** The association between waist-to-calf circumstance ratio (WCR) and cognitive function.

	**Model 1**				**Model 2**			
	***B***	**95% CI (lower)**	**95% CI (upper)**	***P*** **-value**	***B***	**95% CI (lower)**	**95% CI (upper)**	***P*** **-value**
High WCR	−2.194	−2.443	−1.946	<0.001	−0.533	−0.752	−0.315	<0.001
Age					−0.216	−0.227	−0.205	<0.001
Sex					−0.805	−1.028	−0.583	<0.001
Education level					1.051	0.829	1.274	<0.001
Residence					−0.010	−0.384	0.138	0.306
Marital status					0.256	0.022	0.490	0.032
Living arrangement					−0.008	−0.045	0.035	0.322
Occupation before age 60					0.480	0.149	0.810	0.004
Financial support					−0.015	−0.132	0.228	0.083
Total lifestyle score					0.081	0.059	0.103	<0.001
Body mass index					0.027	0.011	0.043	0.001
Physical comorbidities					0.012	−0.224	0.164	0.162
CES-D-10 score					−0.165	−0.187	−0.143	<0.001

Model 1 investigated the association between WCR and cognitive function. Model 2 investigated the association between WCR and cognitive function adjusting for covariates. CES-D-10, The 10-item Center for Epidemiologic Studies Depression. MMSE, mini-mental state examination.

### 3.3. Mediating roles of physical performance and social activity in the association between WCR and cognitive function

**Figure 1:**
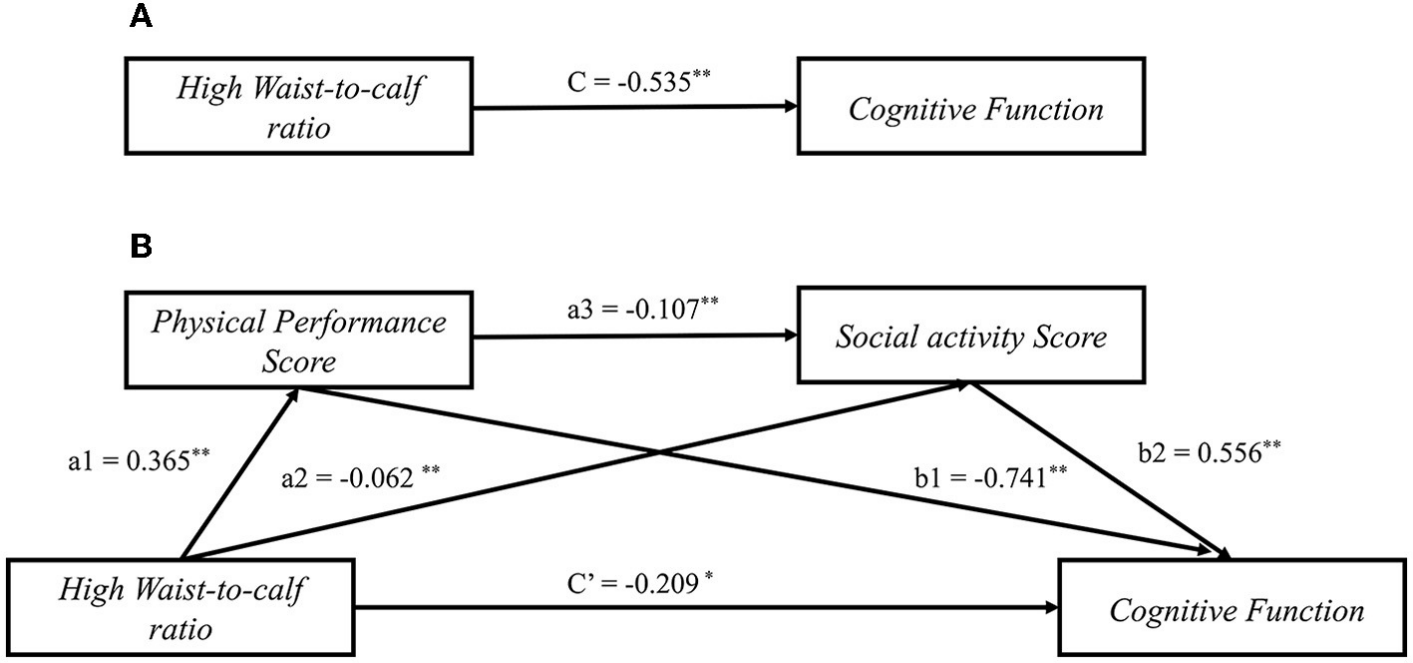
Mediation models of physical performance and social activity for waist-to-calf circumstance ratio on and cognitive function among Chinese older adults. **(A)** The total effect of waist-to-calf circumstance ratio on cognitive function; **(B)** The direct and indirect effects of waist-to-calf circumstance ratio on cognitive function through physical performance and social activity. a1, a2, a3, b1, b2, c', and c represent standardized regression B-coefficients of the path. *P < 0.05, **P < 0.001.

We next sought to clarify the underlying mechanism mediating WCR and cognitive function through physical performance and social activity. The bootstrap results from the mediation analysis are presented in Table 3. Path coefficients and their statistical significance are shown in Figure 1. As shown in Figure 1A, the WCR was negatively correlated with cognitive function (Path c: *B* = −0.535; 95% CI: −0.754, −0.317). There was a positive relationship between the WCR and physical performance score [Path a1: *B* = 0.365; 95% CI: (0.281, 0.449)], that is, a higher WCR value indicates worse physical performance. WCR was negatively associated with cognitive function [Path a2: *B* = −0.062; 95% CI: (−0.096, −0.028)]. Physical performance score had a negative association with cognitive function (Path b1: *B* = −0.741, 95% CI: −0.792, 0.690). Social activity had a significant positive association with cognitive function (Path b2: *B* = 0.556; 95% CI: 0.434, 0.679). The physical performance score had a negative association with social activity (Path a3: *B* = −0.107, 95% CI: −0.115, −0.099). Furthermore, we found that the absolute value of the coefficient of WCR on cognitive function decreased when controlling for physical performance and social activity (Path c': *B* = −0.209, 95% CI: −0.417, −0.011) (Figure 1B).

**Table 3:** The serial mediating effect of waist-to-calf circumstance ratio (WCR) and cognitive function.

**Path**	***B***	**SE**	**95% CI (lower)**	**95% CI (upper)**	***P*** **-value**	**Proportion of effect (%)**
Total effect (c)	−0.535	0.111	−0.754	−0.317	<0.001	100.00
Direct effect (c')	−0.209	0.106	−0.417	−0.011	0.048	39.01
a1	0.365	0.043	0.281	0.449	<0.001	–
a2	−0.062	0.017	−0.096	−0.028	<0.001	–
a3	−0.107	0.004	−0.115	−0.099	<0.001	–
b1	−0.741	0.026	−0.792	−0.690	<0.001	–
b2	0.556	0.063	0.434	0.679	<0.001	–
Total indirect effect	−0.326	0.039	−0.402	−0.251	<0.001	60.93
Indirect effect 1 (a1 × b1)	−0.270	0.035	−0.340	−0.203	<0.001	50.47
Indirect effect 2 (a2 × b2)	−0.035	0.010	−0.055	−0.017	<0.001	6.54
Indirect effect 3 (a1 × a3 × b2)	−0.021	0.004	−0.029	−0.015	<0.001	3.92

Indirect effect 1, high WCR → physical performance → cognitive function; Indirect 2, high WCR → social activity → cognitive function; Indirect3, high WCR → physical performance → social activity → cognitive function. B, standardized regression B-coefficient. SE, standard error. 95% CI, 95% bootstrap confidence interval.

As shown in Table 3, there was a significant correlation between WCR, physical performance, social activity, and cognitive function. WCR had a total effect of −0.535 on cognitive function. In addition, it directly affected SWB by −0.209, accounting for 39.01% of the total effect. The path coefficients of WCR on cognitive function demonstrated that physical performance and social activity had a substantial mediating influence when modeled as mediators (indirect effect 1: a1 × b1 = −0.270, 95% CI: −0.340, −0.203; indirect effect 2: a1 × b1 = −0.035, 95% CI: −0.055, −0.017). Moreover, we found that physical performance and social activity play a serial mediating role in the relationship between WCR and cognitive function (indirect effect 3: a1 × a3 × b2 = −0.021, 95% CI: −0.029, −0.015). As a result, the total mediating effect of physical performance and social activity on WCR and cognitive function was −0.326, accounting for 60.93% of the total effect and involving three types of mediating effects. Physical performance mediated the relationship between WCR and cognitive function by −0.270, which accounted for 50.47% of the total effect. Social activity mediated the relationship between WCR and cognitive function by −0.035, which accounted for 6.54% of the total effect. The serial mediating effect of physical performance and social activity on the association between WCR and cognitive function was −0.021, which accounted for 3.92% of the total effect.

## 4. Discussion

Sarcopenic obesity is a growing public health challenge because of aging populations; however, relevant studies are still in their infancy (Rezende et al., 2019). To the best of our knowledge, this is the first study to examine the relationship between changes in body composition, including a shift toward higher fat mass and decreased lean muscle mass, and cognitive function, as well as the role of social factors, using the indicator of WCR in a sample of older adults aged 65 years or older in China, using a serial chain mediation model. The results showed that high WCR significantly positively predicted cognitive impairment, indicating that older adults with high WCR tended to have decreased levels of cognitive function. Furthermore, the findings confirm that physical performance and social activity partially mediate the relationship between WCR and cognitive function among older adults. Meanwhile, the results also suggest that physical performance and social activity had a significant serial mediation effect on WCR and cognitive function.

The current study confirmed that a high WCR is significantly associated with reduced cognitive function among elderly individuals, which is consistent with previous studies showing that SO or a higher adiposity-to-muscle ratio negatively impacts cognitive function in older adults (Gao et al., 2015, 2020; Ribeiro Santos et al., 2020; Brewster et al., 2021; Chen et al., 2021). This finding suggested that the combination of sarcopenia and obesity could generate a synergetic effect on cognitive impairment rather than a simple superposition, but the mechanism underlying this effect is unclear (Gao et al., 2020). Similarly, the mechanisms underlying obesity-related cognitive dysfunction are still incompletely resolved, although several pathways have been proposed, including sedentary behavior, inflammation, and endothelial function injury (Ryu et al., 2013; Rueger et al., 2016; Roh and Choi, 2020). An even more important predictor of decreased cognition might be sarcopenia or age-related loss of muscle mass and function. Sarcopenia has been linked with poor cognitive function either in epidemiological studies or in experimental *in vitro* and animal studies (Berkman et al., 2000; Bilski et al., 2022). According to a recent meta-analysis, independent of the research population, the definition of sarcopenia, or cognitive impairment, cognitive impairment is related to sarcopenia (Bilski et al., 2022). The role of sarcopenia in cognitive impairment was also confirmed by a neuroimaging study (Schoufour et al., 2021). Accumulated epidemiological, clinical, and basic research evidence indicates that inflammatory markers and the hormonal pathway (e.g., interleukin-6, C-reactive protein, myokine, and serum testosterone) are involved in the association between sarcopenia and cognitive impairment (Shi et al., 2015; Scott et al., 2017; Sharma et al., 2021; Seo et al., 2022). Based on these individual effects, this synergistic effect of sarcopenia obesity on cognition seemed even more apparent, which was supported by a cross-sectional analysis of NHANES data (Shim et al., 2021). Multiple suggested pathogeneses explain the sarcopenia–obesity–cognitive dysfunction link, including chronic inflammation, adipose tissue dysfunction, oxidative stress, insulin resistance, insulin resistance, and mitochondrial dysfunction, all of which are age-related (Snyder et al., 2016; Someya et al., 2022). Moreover, several new mediators have been proposed, such as the muscle–myokine–brain axis and gut–microbiota–brain axis (Spiteri et al., 2019).

Physical performance and social activity were also shown to have partial mediation effects on the relationship between WCR and cognitive function. This finding indicates that the coexistence of reduced lower limb muscle mass with abdominal obesity may impose an ill effect on physical performance and restrict opportunities for elderly individuals to engage in social activities. Recent data suggest that preserving muscular mass (with a larger calf circumference) and avoiding central obesity might help prevent functional impairment even in centenarians (Stern, 2002). Similar to sarcopenia, SO has been linked to frailty and osteoporosis (Fan et al., 2021; Chou et al., 2022). Moreover, individuals with SO are at greater risk of metabolic disorders and reduced physical performance, such as walking speed, than those with sarcopenia or obesity alone (Sun et al., 2021; Tan et al., 2022). It was proposed that individuals with SO tended to suffer from impairment of living functions and lower physical capabilities during aging (Hayes and Preacher, 2014; Jia et al., 2020; Tanaka et al., 2022). As a result of lower physical performance, older adults experience difficulties communicating with others and engaging in daily activities (Tolea et al., 2018; Tanaka et al., 2022). Following the social support theoretical model (Wang H. et al., 2019), this finding suggests that appropriate late-life social activity may buffer the adverse effects of SO on older adults' cognitive function. Multiple studies have discussed the difficulties of participating in social activities for people with SO (Wang R. et al., 2019; Wang et al., 2021; Yang et al., 2022), which accounts for physical activity, oral function, and psychological and nutritional status. Reduced social activity and limited social interactions imply restricting social connections, which may result in the development of cognitive decline (Sun et al., 2021; Yang et al., 2021; Yin et al., 2021). In addition, we found that physical performance was a stronger mediator than social activity, which might not be adequately explored in previous research (Gao et al., 2020; Ribeiro Santos et al., 2020).

Our study's main result is that physical performance and social activity operate as a series of intermediaries in the link between WCR and cognitive function. Based on the theory of cognitive reserve (Yin et al., 2019), cognitive stimulation from social interactions may promote better cognitive aging (Yue et al., 2022). This result is consistent with previous studies and extends them by demonstrating the serial intermediating effect of physical performance and social activity in the association between SO and cognitive function among older adults (Brewster et al., 2021; Shim et al., 2021). The observed serial mediation role in the present study might be attributed to several behavioral mechanisms, all of which can increase the risk of cognitive impairment (Zeng, 2012). First, an increase in fat mass and a decrease in muscle mass are distinct factors that contribute to disability, incapacity, and mortality (Choe et al., 2018). Furthermore, it appears that SO has a synergistic effect on physical performance in older adults (Zeng et al., 2017). Second, the deterioration of the physical performance of elderly individuals increases their difficulty in participating in social activities. There was an independent association between physical frailty and all social activities (Zhong et al., 2016). Third, higher levels of social activity are associated with greater cognitive reserve, which results in the activation and strengthening of various neural circuits and behavioral pathways, improving the ability to compensate for adverse structural and functional brain consequences caused by hearing loss or other sensory impairments (Zhong et al., 2017; Kokkeler et al., 2019). In contrast, social isolation correlates with both restructuring and functional changes in the brain's social network and in brain regions that are related to mentalizing and social interaction, according to the social brain hypothesis (Zhuang et al., 2022). Given the positive effects of close social ties on health behavior, social interaction may influence cognitive outcomes (social control hypothesis) (Zovetti et al., 2021). Overall, maintaining physical function and participation in social activities may lessen the negative effects of high WCR on cognitive function in older adults.

Some limitations should be addressed in this study. First, the cross-sectional design renders causal inferences about the association between WCR, physical performance, social activity, and cognitive function, which could be explored in the future with a longitudinal design. Second, although geographically broad, the sample is not a random sample of the Chinese geriatric population. Third, self-report measures can be prone to bias and distortion. It is, therefore, essential to use multiple measures, such as an in-depth interview or observation of behavior. Finally, since we lacked muscle strength measurements, we used a surrogate marker for SO that only considers low muscle mass and obesity, not muscle function in this study. Although there is no consensus on the definition of SO, which varies considerably, we think this information should be taken into account in future research.

Despite the aforementioned limitations, there are some practical implications to our findings. Around the world, we must change the way we look at sarcopenic, obesity, and how SO impacts the physical, social, and cognitive functioning of older adults. From a practical view, given that high WCR can decrease the cognitive function of elderly individuals, families, caregivers, healthcare personnel, and institutions should pay more attention to old adults with SO or high-risk groups. First, prevention of SO rather than its treatment is more rational since attempting to reverse age-related diseases among the elderly is difficult due to their general disability, as well as their unwillingness to modify lifestyles and adhere to long-term medications. Unfortunately, SO among older adults has not received sufficient attention in all walks of life. In particular, age-related SO remains underdiagnosed and untreated, despite evidence suggesting that treatment (e.g., lifestyle intervention) can mitigate adverse outcomes (Snyder et al., 2016). Indeed, the patients themselves do not consider it a serious health matter either, treating SO as a normal aging phenomenon rather than a multifactorial disease. Although a cliché, early screening, diagnosis, and intervention should be performed. The identification of vulnerable individuals is essential to ensure that prevention and early intervention programs are targeted at them. In rehabilitative practice, broader consultations could specifically include discussing emotional aspects of social interaction with patients and how SO affects cognitive and physical functioning. This serial mediation model has the potential to facilitate earlier identification and increase motivation for SO diagnosis and treatment, as well as prevention. Overall, this model could be beneficial for older adults with SO, their families, and social circles, the healthcare system, and society as a whole. Currently, there is no approved pharmacological treatment for SO, regardless of novel drugs under investigation. As a result, the current management of SO focuses on weight loss and increased physical activity (Rezende et al., 2019). A growing body of evidence indicates that the addition of exercise to diet adjustment can increase myokine release from tissues into the blood and delay the onset and progression of SO, which has the potential to influence protein metabolism, mitochondrial quality control, inflammation, and other processes (Spiteri et al., 2019).

## 5. Conclusion

Overall, we discovered that physical performance and social activity serve as a series mediator in the relationship between WCR and cognitive function in a nationally representative sample of older Chinese adults. It might be worthwhile to recommend multidimensional health and social interventions aimed at improving physical and cognitive function as well as social inclusion among older adults with SO. Better levels of physical performance and social activity are connected with higher levels of cognitive function, with physical performance having a stronger influence than social activity. The link between high WCR and cognitive impairment implies that more focused treatments should be implemented to improve cognitive and physical performance in older adults with SO. Furthermore, authorities should focus on physical performance recovery and encourage older persons with SO to engage in social activities according to their health status.

## Data availability statement

The datasets presented in this study can be found in online repositories. The names of the repository/repositories and accession number(s) can be found below: https://opendata.pku.edu.cn/dataverse/CHADS.

## Author contributions

XC and JZ conceived the concept and design of the study. XC and BY contributed to data cleaning and analysis. BY and JZ contributed to the writing assistance and proofreading of the article. All authors approved the final version of the manuscript.
