# Supplementary material for: Waist-to-calf circumstance ratio and cognitive function among Chinese older adults: Mediating roles of physical performance and social activity
Source: Front Aging Neurosci. 2023 Apr 17;15:1166341. doi: 10.3389/fnagi.2023.1166341 (PMC10150408; doi:10.3389/fnagi.2023.1166341)
Supplement: Supplementary file 1 [file Table_1.docx]

**Supplementary Material**

- **Table S1 Description of study variables**
- **Table S2 The Chinese version of the mini-mental state examination (MMSE) in the CLHLS**
- **Table S3 The coding of physical performance**
- **Table S4 The coding of social activity score**
- **Table S5 The coding of lifestyle score**

- **Table S6 The coding of the 10-item Center for Epidemiologic Studies Depression (CES-D-10)**

**Table S1. Description of study variables**

| Variables | Description |
| --- | --- |
| **Independent variables** |  |
| High WCR | 0 = No, 1 = Yes |
| **Dependent variables** |  |
| Cognitive function | MMSE score (Range: 0-30) |
| **Mediator** |  |
| Physical performance | Physical performance score (Range: 0-8) |
| Social activity | Social activity score (Range: 0-3) |
| **Covariates** |  |
| Age | Validated age of the sample |
| Gender | 0 = Male, 1 = Female |
| Education level | 0 = Illiterate, 1 = Literate |
| Residence | 0 = Rural, 1 = Urban |
| Marital status | 0= Other statuses, 1 = Currently married and living with spouse |
| Living arrangement | 0 = Living alone, 1 = Living with someone else |
| Occupation before age 60 | 0 = Non-professional work, 1 = Professional work |
| Financial support | 0 = Insufficient, 1 = Sufficient |
| Lifestyle | Total Lifestyle score (Range: 10-50) |
| Physical comorbidities | 0 = No, 1 = Yes |
| Depressive symptom | CES-D-10 (Range: 0-30) |

WCR, Waist-to-calf Circumstance Ratio. MMSE, Mini-Mental State Examination. CES-D-10, The 10-item Center for Epidemiologic Studies Depression.

| **Table S2. The Chinese version of the mini-mental state examination in the CLHLS (score range: 0-30)** | | |
| --- | --- | --- |
| **Domains** | **Questions** | **Scores** |
| Orientation | What time of day is it right now (morning, afternoon, evening)? | 1 |
|  | What is the month (Western or Chinese calendar) right now? | 1 |
|  | What is the date (Chinese calendar day and month) of the mid-autumn festival? | 1 |
|  | What is the season right now, spring, summer, fall, winter? | 1 |
|  | What is the name of this district or town? | 1 |
|  | Please name as many kinds of food as possible in 1 minute. | 7 |
| Registration | repeat table, apple and clothes | 3 |
| Attention and calculation | I will ask you to spend 3 dollars from 20 dollars, then you must spend 3 dollars from the number you arrived at and continue to spend 3 dollars until you are asked to stop. | 5 |
|  | Asking the interviewee to copy a figure, in which all the sides and angles are correct | 1 |
| Recall | repeat the three words (in any order) that you heard a little while ago | 3 |
| Language | Naming pen and watch. | 2 |
|  | Repeating the following sentence: “What you plant, what you will get.” | 1 |
|  | The individual is asked to take a paper using right hand, fold it in the middle using both hands, and place the paper on the floor. | 3 |

| **Table S3. The coding of** **physical performance (score range: 0-8)** | | |
| --- | --- | --- |
| **Domains** | **Questions** | **Scores** |
| Muscle strength | Are you able to lift 5 kg of weight? | 0 = without problem, 1 = with problem, 3 = not able to  0 = without problem, 1 = with problem, 3 = not able to  0 = without problem, 1 = with problem, 3 = not able to  0 = without problem, 1 = with problem, 3 = not able to |
| Walking ability | Are you able to walk 1 km? |  |
| The strength of the lower extremities | Are you able to crouch and stand three times? |  |
| Core strength | How do you stand up after sitting in a chair? |  |

| **Table S4. The coding of social activity score (score range: 0-3)** | |
| --- | --- |
| **Variable** | **Coding** |
| Engagement in play cards/mah-jongg | 0=never; 1= almost every day /not daily, but once for a week/not weekly, but at least once for a month/not monthly, but sometimes |
| Engagement in organized activities | 0=never; 1= almost every day /not daily, but once for a week/not weekly, but at least once for a month/not monthly, but sometimes |
| Visiting experiences | 0=never; 1= almost every day /not daily, but once for a week/not weekly, but at least once for a month/not monthly, but sometimes |

| **Table S5. The coding of lifestyle score (score range: 10-50)** | |
| --- | --- |
| **Variable** | **Coding** |
| **Lifestyle score (score: 10-50)** |  |
| **Dietary pattern score (score: 7-38)** |  |
| The staple food pattern (score: 0-5) | 0= did not take corn as staple food; 1-5= took corn according to the quintile ranking of daily consumption  the quintile ranking of daily consumption month/not monthly, but sometimes |
| Fruit intake (score: 1-4) | 1-4= rarely or never/occasionally/quite often/every day or almost/every day  never /not daily, but once for a week/not weekly, but at least once for a month/not monthly, but sometimes |
| Vegetable intake (score: 1-4) | 1-4= rarely or never/occasionally/quite often/every day or almost/every day |
| Fish intake (score: 1-5) | 1-5= rarely or never/occasionally/at least once per month/ at least once per week/almost every day  week /every day |
| Milk intake (score: 1-5) | 1-5= rarely or never/occasionally/at least once per month/ at least once per week/ almost every day  week /every day |
| Nut intake (score: 1-5) | 1-5= rarely or never/occasionally/at least once per month/ at least once per week/almost every day  week /every day |
| Meat intake (score: 1-5) | 1-5= almost every day /at least once per week/ at least once per month/ occasionally/ rarely or never |
| Sugar intake (score: 1-5) | 1-5= almost every day /at least once per week/ at least once per month/ occasionally/ rarely or never |
| **Daily life habit scores (score: 3-12)** |  |
| Tobacco use (score: 1-4) | 1-3=had the third, second, and first tertile amount of smoking times per day; 4= did not smoke  amount of smoking times per day; 1= spouse/so |
| Alcohol consumption (score: 1-3)  consumption | 1-3= daily alcohol consumption in the third tertile/in the middle tertile /in the first tertile |
| Performed outdoor activities (score: 1-5) | 1-5=never/not every month but sometimes /not every week but at least once a month/not every day but at least once a week/  but at least once a week/almost every day |

| **Table S6. The coding of** **the 10-item Center for Epidemiologic Studies Depression (score range: 0-30)** | |
| --- | --- |
| **Questions** | **Scores** |
| Are you worried about some small things? | 0 = rarely, 1 = some days, 2 = occasionally, 3 = most of the time  0 = rarely, 1 = some days, 2 = occasionally, 3 = most of the time  0 = rarely, 1 = some days, 2 = occasionally, 3 = most of the time  0 = rarely, 1 = some days, 2 = occasionally, 3 = most of the time |
| Is it difficult to concentrate when you are doing things now? |  |
| Are you feeling sad or depressed? |  |
| Do you think that the older you are, the less useful you are, and the hard work? |  |
| Are you full of hope for future life? | 0 = most of the time, 1 = occasionally, 2 = some days, 3 = rarely |
| Are you nervous and scared? | 0 = rarely, 1 = some days, 2 = occasionally, 3 = most of the time |
| Do you feel as happy as you are when you are young? | 0 = most of the time, 1 = occasionally, 2 = some days, 3 = rarely |
| Do you feel lonely? | 0 = rarely, 1 = some days, 2 = occasionally, 3 = most of the time |
| Do you feel unable to continue your life? | 0 = rarely, 1 = some days, 2 = occasionally, 3 = most of the time |
| How is your sleep quality now? | 0 = rarely, 1 = some days, 2 = occasionally, 3 = most of the time |
